# Atmospheric CO_2_ during the Mid-Piacenzian Warm Period and the M2 glaciation

**DOI:** 10.1038/s41598-020-67154-8

**Published:** 2020-07-09

**Authors:** Elwyn de la Vega, Thomas B. Chalk, Paul A. Wilson, Ratna Priya Bysani, Gavin L. Foster

**Affiliations:** School of Ocean and Earth Science, University of Southampton, National Oceanography Centre Southampton,Waterfront Campus Southampton, Southampton, SO14 3ZH UK

**Keywords:** Climate sciences, Ocean sciences

## Abstract

The Piacenzian stage of the Pliocene (2.6 to 3.6 Ma) is the most recent past interval of sustained global warmth with mean global temperatures markedly higher (by ~2–3 °C) than today. Quantifying CO_2_ levels during the mid-Piacenzian Warm Period (mPWP) provides a means, therefore, to deepen our understanding of Earth System behaviour in a warm climate state. Here we present a new high-resolution record of atmospheric CO_2_ using the δ^11^B-pH proxy from 3.35 to 3.15 million years ago (Ma) at a temporal resolution of 1 sample per 3–6 thousand years (kyrs). Our study interval covers both the coolest marine isotope stage of the mPWP, M2 (~3.3 Ma) and the transition into its warmest phase including interglacial KM5c (centered on ~3.205 Ma) which has a similar orbital configuration to present. We find that CO_2_ ranged from ${{\bf{394}}}_{{\boldsymbol{-}}{\bf{9}}}^{{\boldsymbol{+}}{\bf{34}}}$ ppm to ${{\bf{330}}}_{{\boldsymbol{-}}{\bf{21}}}^{{\boldsymbol{+}}{\bf{14}}}$ ppm: with CO_2_ during the KM5c interglacial being ${{\bf{391}}}_{{\boldsymbol{-}}{\bf{28}}}^{{\boldsymbol{+}}{\bf{30}}}$ ppm (at 95% confidence). Our findings corroborate the idea that changes in atmospheric CO_2_ levels played a distinct role in climate variability during the mPWP. They also facilitate ongoing data-model comparisons and suggest that, at present rates of human emissions, there will be more CO_2_ in Earth’s atmosphere by 2025 than at any time in at least the last 3.3 million years.

## Introduction

The Pliocene Epoch (2.588 to 5.3 Ma) was a time when global temperatures were ~3 °C warmer than the pre-industrial^1^ and sea level was ~20 m higher than present^2,3^, largely due to the presence of smaller Greenland and Antarctic ice sheets^2^. Given that many other tectonic boundary conditions were similar (but not identical^4^) to today, the Pliocene Epoch, and the mid-Piacenzian warm period (mPWP; 3 to 3.3 Ma) in particular, are useful targets for climate model validation studies (e.g. refs. ^4–7^). Phase 1 of the Pliocene Model Intercomparison Project (PlioMIP), found an overall agreement between climate model simulations of mPWP surface temperatures and the available data when run with a CO_2_ of 405 ppm^1^. The North Atlantic and Pacific Oceans, however, are areas of consistently poor data-model agreement^1,6^. Haywood *et al*.^4^ noted that the experiment design of PlioMIP simulations precluded a determination of whether this result is attributed to poor model performance or poor data quality. This deficiency in experimental design, in part, resulted from the data collection time interval being from 3 to 3.3 Ma which spans a number of orbital climate cycles, whereas the models were run for less than 1,000 years with an invariant modern orbit^5^.

**Figure 1:**
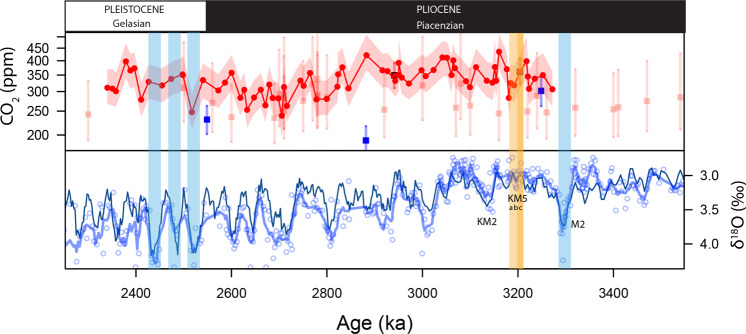
Top panel: Current CO_2_ estimates from boron isotopes across the Plio-Pleistocene boundary. *G. ruber* data in red circles from site 999 (Martinez-Boti *et al*.^13^), *T. sacculifer* in red squares from site 999 (Seki *et al*.^14^), blue squares from site 926 (Sosdian *et al*.^20^) and pale red squares from site 999 (Bartoli *et al*.^12^). Bottom Panel: δ^18^O from benthic foraminifer *Cibicidoides wuellerstorfi* at ODP Site 999 (blue circles) with a 5 point running mean (this study and ref. ^53^) compared to the benthic isotope stack of ref. ^19^. M2 glacial and early Pleistocene strong glacials are highlighted in blue for context. Interglacial KM5 and KM5c are highlighted in yellow and orange, respectively. Note that there are no estimates for the M2 glacial and very few across the mid-Piacenzian warm period (mPWP), the low resolution of previous studies makes pin-pointing individual interglacials such as the KM5c future analogue difficult. These studies also differ in their estimates of Mid-Piacenzian CO_2_^12–14,20^.

To address this weakness, PlioMIP2 (phase 2)^5^ part of the model evaluations^8^ feeding into the 6^th^ Assessment Report (AR) for the Intergovernmental Panel on Climate Change IPCC AR6, will focus on the KM5c interglacial interval at ~3.205 Ma which has a close-to-modern orbital configuration^4^. Other data compilation efforts with future data-model comparisons in mind, have targeted the interval from 3.3 Ma to 3.205 Ma^1,9^ because this also includes marine isotope stage M2, a prominent anomalously cold marine isotope stage (MIS) that provides a cold-to-warm transition within the overall warm background climate state of the late Pliocene (Fig. 1). A major limitation of these latest efforts, however, is the lack of data on atmospheric CO_2_ during these rather narrow time intervals^9–11^. For instance, although ~40 δ^11^B-based determinations of CO_2_ are available for the 3 to 3.3 Ma window^12–14^, the M2 to KM5 transition remains poorly sampled and disagreement between datasets from different analytical techniques persist (Fig. 1). Furthermore, the available δ^11^B-CO_2_ data exhibit relatively large short-term variability, which could signal orbital cyclicity (41 kyrs) aliased by low sampling resolution. Data from other CO_2_ proxy systems such as stomata and palaeosol δ^13^C, are lacking in this interval and it has recently been shown that the marine-based alkenone-δ^13^C-CO_2_ proxy underestimates CO_2_ levels in the Pliocene^15,16^ therefore limiting its usefulness to providing a minimum CO_2_ during the mPWP of >270 ppm^16^.

To address this data deficiency, we developed δ^11^B-based CO_2_ estimates from Ocean Drilling Program (ODP) Site 999 in the Caribbean (Supplementary Fig. 1) at a resolution of 1 sample per 3–6 kyr for the time interval 3.15 to 3.35 Ma, encompassing the M2 glaciation and the KM5 interglacial (including KM5c). Although focusing predominantly on the mixed layer dwelling planktic species *Globigerinoides ruber* (45 new data points, 63 in total), we also present new measurements of *Trilobus sacculifer* (2 new data points, 5 total) on the same samples to provide a check on the consistency of the δ^11^B-pH calibration for *G. ruber* which has been recently called into question^17^.

## Results

**Figure 2:**
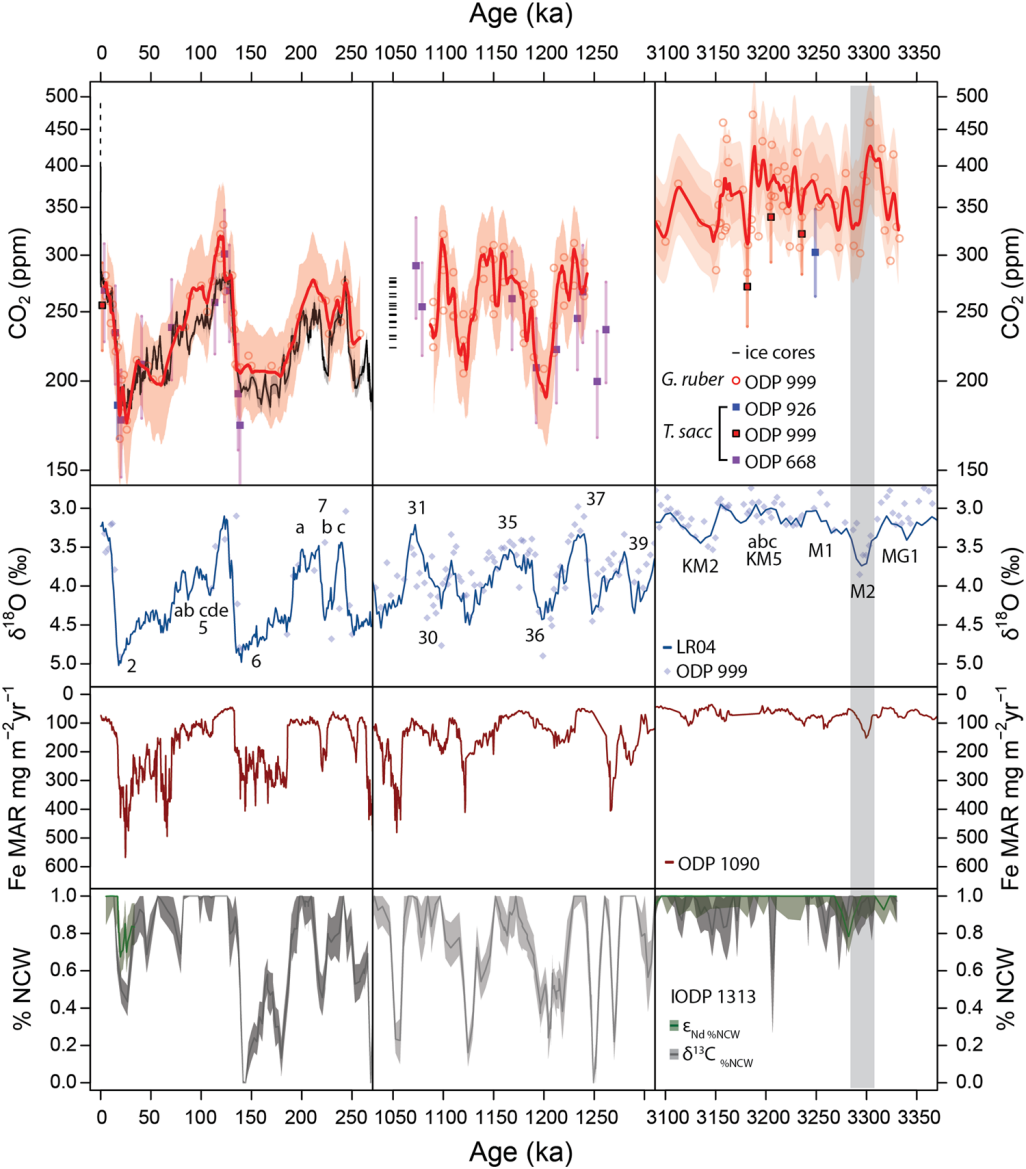
Top panel: Red circles and lines show δ^11^B-derived CO_2_ data from *Globigerinoides ruber* at ODP Site 999 (this study and Martinez-Boti *et al*.^13^, Chalk *et al*.^18^), red squares are *Trilobatus sacculifer* at ODP 999 (this study and Seki *et al*.^14^), purple squares are *T. sacculifer* from ODP 668 (Honisch *et al*.^23^) and blue squares are *T. sacculifer* from ODP 926 (Sosdian *et al*.^20^). Black solid line shows ice core-derived CO_2_ from ref. ^58^. Left; Late Pleistocene CO_2_ from boron isotopes^14,18,23,52^ and ice core data. Also shown are CO_2_ projections in line with RCP8.5 at current emission rates to the year 2040 (black broken line). Middle column; MPT CO_2_ estimates^18,23^ including disturbed ice estimates^24,25^ (Note: age adjusted for scale). Right; mPWP estimates of CO_2_ (this study combined with Martinez-Boti *et al*.^13^), new data from *T. sacculifer* is shown in red squares and shows no offset from *G. ruber* estimates. Second panel: Time periods as above, LR04 and ODP 999 δ^18^O from *C. wuellerstorfi*^18,19,53^. Third panel: Iron mass accumulation rate from the Southern Atlantic ODP Site 1090^28^. Fourth panel: % Northern Component Water (NCW) estimated from δ^13^C in benthic foraminifera (grey) and ɛ_Nd_ from fish debris (dark green) in the deep North Atlantic (core U1313^31^). Note the lag of ocean circulation and CO_2_ relative to the M2 glaciation^31^.

Our new high-resolution CO_2_ record is shown in Fig. 2 (and Supplementary Fig. 2 and Supplementary Table 2) and is consistent with earlier studies^12–14^ in showing that CO_2_ was higher than the pre-industrial during the mPWP (mean=360 ppm). Our more detailed record reveals that CO_2_ variations ranged from ${330}_{-21}^{+14}$ to ${394}_{-9}^{+34}$ ppm (based on the mean and distribution of CO_2_ in the <25% and >75% interquartile range during the whole studied period; ref. ^18^), with a peak-to-trough range of 56–75 ppm determined by a Welch T-test of the data within the upper and lower quartiles (at 95% confidence; p < 0.01). From a comparison with benthic δ^18^O data from ODP 999 (Supplementary Table 2) and the δ^18^O stack^19^ (LR04) we observe that cold marine isotope stages (e.g. KM2; Fig. 2) are typically closely associated with low CO_2_ and warm stages with high CO_2_ levels. However, during the prominent M2 cold stage and through the warming out of M2, CO_2_ appears to lag benthic δ^18^O by ~10 kyr (Supplementary Fig. 5). This lag is not attributable to age model uncertainty because it is present when comparing δ^11^B-derived CO_2_ and benthic δ^18^O from the same samples (Fig. 2). CO_2_ during the interglacial KM5c, determined using the mean of the δ^11^B of the five samples in this interglacial, is estimated at ${391}_{-28}^{+30}$ ppm (at 95% confidence). Using a broader time window (10 to 15 ky) for the KM5 interglacial moderately alters the estimate (see Supplementary Table 1).

Our estimates of borate ion, pH and CO_2_ derived from the δ^11^B of *T. sacculifer* (without sac-like final chamber) from ODP 999 (our new data, and ref. ^14^) and ODP 926^20^ overlap well with those based on *G. ruber* from ODP 999 in the mPWP (Fig. 2 and Supplementary Fig. 3). Calculated CO_2_ does not exhibit substantive inter-species offset with a mean difference of 10 ± 29 ppm with no consistent bias towards higher or lower pH/CO_2_.

### Accuracy of δ^11^B-CO_2_ from *G. ruber* in the Plio-Pleistocene

Ref. ^17^ suggested that the δ^11^B-pH calibration of *G. ruber* may have evolved through time and that *G. ruber* may suffer from morphotype-differences in δ^11^B-derived pH estimates for the surface Pliocene ocean resulting in underestimates of the true pH and corresponding overestimates of true CO_2_. The principal evidence presented for this assertion was the disagreement between Pliocene CO_2_ calculated using the *T. sacculifer* data of Bartoli *et al*.^12^ and the *G. ruber* data from Martinez-Boti *et al*.^13^ (Fig. 1). As shown here, when *T. sacculifer* and *G. ruber* are compared from the same samples and measured with the same analytical technique (in this case MC-ICPMS) there is no significant offset between the methods in terms of reconstructed borate ion δ^11^B, pH or CO_2_ (Fig. 2 and Supplementary Fig. 3). This finding suggests that the *G. ruber* δ^11^B-pH calibration has not evolved through time and therefore that the pH (and hence CO_2_) we reconstruct here is an accurate record of the surface water carbonate system parameter. This finding indicates that disagreements between published Pliocene δ^11^B-based datasets^12,13,20,21^ are likely attributable to either: (i) sampling driven aliasing due to the relatively large short-term CO_2_ variability in the mPWP (e.g. Figure 2), (ii) differences in core site location between studies (with possible different local CO_2_ disequilibrium), (iii) the lack of a comparison between species on exactly the same sample, or (iv) the well-documented analytical offset between MC-ICPMS and negative-ion thermal ionization mass spectrometry (NTIMS; see refs. ^21,22^). We note that, if the offset is attributable to analytical issues, the agreement between data generated by both methodologies for the younger Pleistocene time slices examined here (Fig. 2) confirms the suggestion of Sosdian *et al*.^20^ that the *T. sacculifer* dataset of Bartoli *et al*.^12^ (measured with NTIMS) requires an additional analytical correction (see ref. ^16^ for details), beyond that currently used for the NTIMS δ^11^B datasets of refs. ^17,23^.

### mPWP CO_2_ cycles – variability and causes

**Figure 3:**
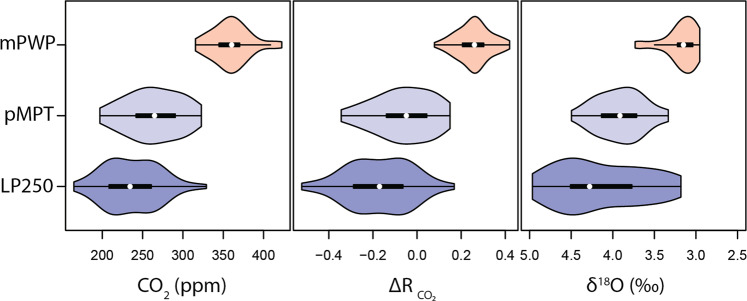
Distribution plots showing the distribution of absolute CO_2_ (left), climate forcing from CO_2_ (middle), and δ^18^O (right) across three time intervals (where CO_2_ cycles are consistent); Late Pleistocene 0–250 ka (LP250, bottom), Mid-Pleistocene (MPT, 1050–1500 ka) and Middle Piacenzian including M2 (mPWP, 3–3.3 Ma). The white dots represent the median values with the quartile ranges shown in the thick and thin black lines. The width of the distribution shows the density of data in each range. Climate forcing (ΔR_CO2_) is expressed relative to pre-industrial CO_2_ value of 278 ppm. The effect of excluding M2 in the mPWP interval has a negligible effect on the distribution of CO_2_ and CO_2_ forcing but a visible impact on δ^18^O.

Our new high-resolution data set clearly demonstrates that the mPWP is an interval of relatively high CO_2_, with a mean of 367 ppm whereas lower values are observed during the mid- and late-Pleistocene (262 ppm and 241 ppm^18^, respectively Fig. 2). Our dense sampling permits, for the first time, an assessment of CO_2_ variability during mPWP on orbital timescales, although the length of our record still precludes reliable time series analysis. The CO_2_ cycles (and derived climate CO_2_ forcing) we observe in the mPWP (including the M2 glaciation) are similar, yet smaller in amplitude, than those evident in δ^11^B-CO_2_ data from the Late Pleistocene (LP = 0–250 kyr; ref. ^18^) and early mid-Pleistocene Transition (eMPT = 1050–1250 kyr; Figs. 2 and 3). The variation in climate forcing between these different intervals correlates with the magnitude of δ^18^O variability, although there is an apparent increased response in δ^18^O during the late Pleistocene (δ^18^O range ~ 2‰) relative to the mPWP (δ^18^O range ~ 0.5–1‰ Fig. 3; ref. ^13^) likely reflecting the increased influence of ice-volume change on δ^18^O following the northern hemisphere glaciation^13^.

We also compare the δ^11^B-derived CO_2_ data with ice-core based CO_2_ for the Pleistocene intervals^18,23^ (Supplementary Fig. 4). We expect the range of CO_2_ variability from the various Antarctic ice cores to be narrower than our marine-based records because of the greater precision associated with the ice core records (± 6 ppm vs. ±20–30 ppm) and the CO_2_ data from blue ice from the mid-Pleistocene may not capture a full climate cycle^24,25^. Despite these caveats, and as discussed elsewhere^18,25^, there is good agreement between the ice-core and δ^11^B-derived CO_2_, providing confidence in the accuracy of the distribution (and absolute values) we determine here for the mPWP.

Three phenomena are suggested to exert a major control over glacial-interglacial CO_2_ change in the Late Pleistocene record: (*i*) changes in stratification south of the Antarctic Polar Front influencing the ventilation of CO_2_-rich circumpolar deep water;^26^ (*ii*) variations in the magnitude of dust-borne Fe fertilization in the sub-Antarctic that influences the strength of the biological pump in this region;^27,28^ (*iii*) changes in the extent to which southern component deep water fills the North Atlantic increasing deep ocean carbon storage^29^. Ref. ^30^ proposed that polar waters in the North Pacific and the entire Southern Ocean (SO) were destratified prior to the onset of Northern Hemisphere Glaciation at 2.7 Ma. This mechanism may contribute to elevated CO_2_ during the mPWP^30^, but it would also rule out water mass ventilation in the high-latitude SO as an important driver of the CO_2_ cycles we observe in the mPWP. Both the accumulation and variability of dust-borne Fe at ODP Site 1090 (Fig. 2) were reduced during the mPWP to a fraction of that observed in the other time intervals examined (~ factor of four reduction during the mPWP, compared to the late Pleistocene), suggesting that dust supply did not play a major role in generating the observed CO_2_ cycles in the mPWP.

Chemical stratification of the North Atlantic Ocean, due to incursions of southern component water (SCW), during the mPWP is suggested by gradients in ɛ_Nd_ and δ^13^C from ref. ^31^, albeit at reduced magnitude compared to the latest Pleistocene and eMPT. Ref. ^31^ showed that the SCW-signal was particularly marked after the M2 glacial maximum as shown by reduced % NCW (northern component water, Fig. 2), and also observed that ɛ_Nd_ lagged δ^18^O by 10 kyr during the onset of the M2 glaciation, as is also evident in our new CO_2_ data (Fig. 2, and supplementary Fig. 5). In general, those intervals of the mPWP where SCW contributed significantly to the waters of the deep North Atlantic were characterized by low CO_2_ (and vice versa; Fig. 2), yet with variable leads and lags (supplementary Fig. 5). This association is consistent with the suggestion^31,32^ that glacial expansion of a CO_2_-charged SCW reservoir plays at least a first order role in driving orbital CO_2_ cycles, even prior to the onset of northern hemisphere glaciation at 2.7 Ma.

### M2 Glaciation – the role of CO_2_

Marine Isotope Stage M2 (at 3.3 Ma) is an anomalously cold stage clearly evident in the LR04 δ^18^O stack^19^ and many other temperature records, such as arctic air temperature^33^ and sea surface temperature^34,35^ (including site ODP 999, supplementary Fig. 6) It is also often described as a failed attempt at Northern Hemisphere Glaciation^34,36,37^, that eventually initiated ~600 kyr later at ~2.6 Ma. Refs. ^13,14^ used δ^11^B-CO_2_ to suggest that CO_2_ dropped below 280 ppm for the first time during the intensification of Northern Hemisphere Glaciation (iNHG), consistent with the suggestion that 280 ppm CO_2_ is an important threshold  below which extensive glaciation of continents in the northern hemisphere develop^38^. Our new data reveal that while the lower bound of the error envelop in our smoothed CO_2_ record approaches this value, atmospheric CO_2_ during M2 is unlikely to have crossed this threshold. Furthermore, in our records, CO_2_ variability associated with M2 lags δ^18^O by 10 kyr, which also means that minimum CO_2_ is not coincident with minimum northern hemisphere orbital forcing (Supplementary Fig. 5). Additional support for CO_2_ playing a secondary role in M2 is that other periods of low CO_2_ are evident in the mPWP (Fig. 2). For instance, the KM2 glaciation is clearly evident in the benthic δ^18^O data, Mg/Ca-SST at site 999 (Fig. 2 and Supplementary Fig. 6) and our CO_2_ record, but is not considered to be a major glacial interval^19^ (Fig. 2). This therefore suggests that other boundary conditions, such as orbital configuration^39^ (Supplementary Fig. 5) were perhaps dominant in triggering the M2 cold stage.

### M2 Glaciation - CO_2_ lags δ^18^O

Minor dephasing between ɛ_Nd_, δ^13^C and benthic δ^18^O has been observed in the late Pleistocene^40^, with carbon budget change lagging δ^18^O and preceding the change in ocean circulation, but with smaller lags than we observe during M2 (~2.5 ky vs. ~10 ky).

Similarly, while variations in atmospheric CO_2_ are not entirely in phase with ice volume changes over the late Pleistocene, the leads-lags are small^41^ (<3 ky) and are not readily observed when comparing δ^11^B-derived CO_2_ and benthic foraminiferal δ^18^O time series (e.g. Figure 2 left panels).

This therefore either requires the operation of subtly distinct carbon-cycle dynamics during the Pliocene, and M2 in particular, or implies some other driver operated to explain the observed ~10 kyr lag of CO_2_ during M2.

One possible non-carbon cycle driver for the observed lag at M2 could be a preservation bias in our data because partial dissolution of planktic foraminiferal tests drives δ^11^B towards more negative isotopic composition (lower pH, higher CO_2_) in some species^14,42^. However, while our chosen species for pH/CO_2_ reconstruction, *G. ruber*, is known to be relatively susceptible to partial dissolution on the seafloor^43^, its δ^11^B signal has been observed to be robust^14,44^. Furthermore, a recent study^45^ of test weight and fragmentation at ODP 999 showed that tests become better preserved during M2 (Supplementary Fig. 7), as observed during glacial periods of the late Pleistocene^46^. This is inconsistent with the high CO_2_ observed during the descent into M2 maximum (especially given fragmentation and δ^18^O are in phase, Supplementary Fig. 7), ruling out an effect of partial dissolution on our CO_2_ reconstruction and observed lag.

An alternative explanation for the delayed pH change observed at ODP 999 during M2 lies in local water mass changes during this glacial episode. Although the Central American Seaway was closed to deep water by this time^36^, it is possible that limited exchange of surface water was still occurring around M2^34^ and the early Pleistocene^47^. Temperature data from Mg/Ca in *T. sacculifer*^34^ and *G. ruber* at site 999 (this study, Supplementary Fig. 6, data from the East Equatorial Pacific site 1241^48^ is also shown for comparison) show a cooling prior to the M2 maximum. A connection between the East Equatorial Pacific and the Caribbean, or local intensification in upwelling could have brought cold, nutrient- and carbon-rich waters (low Mg/Ca, low pH) to Site 999, potentially explaining the apparent high CO_2_ at the inception of M2. However, because no noted decline in the abundance of *G. ruber* occurred, given this is a species known to favour oligotrophic conditions^49^, it seems unlikely this was accompanied by a significant influx of such water. Also, a mechanism for increased influx of EEP water during sea level regression is lacking. Importantly, during KM5c (3205ky), the CAS likely remained closed impeding the influx of Pacific waters to site 999, as shown by elevated temperature (relatively to the M2 interval) in the Caribbean (Supplementary Fig. 6), hence local changes in hydrography are unlikely to have unduly impacted our CO_2_ estimates for this central interval.

The cause of the apparent lag of CO_2_ compared to δ^18^O during the inception of M2 therefore remains uncertain, but because this lag is also present in North Atlantic ɛ_Nd_ records^31^ we favour a carbon cycle-based interpretation. A Southern Ocean-driven explanation for the CO_2_ lag during M2 is perhaps indicated by the observation that the tail of the CO_2_ decline during M2 is out of phase with decline in northern hemisphere insolation, but apparently in-phase with insolation decline at 65°S (i.e. offset by one half precession cycle, Supplementary Fig. 5).

### Past to future – when will we exceed mPWP CO_2_ levels?

Atmospheric CO_2_ has been increasing since the industrial revolution from a background of ~280 ppm, reaching 411 ppm in 2019. The mPWP is often cited as being the last time CO_2_ levels were as high as today^13^, although we note methane and other greenhouse gases remain poorly constrained and may contribute to extra radiative forcing^50^.

Our refined view of CO_2_ during this interval however reveals that in terms of the mean (367 ppm), mPWP values were exceeded in the mid-1990s. Our upper quartile range (${394}_{-9}^{+34}$ ppm) suggests that CO_2_ during the mPWP *is very likely to have been* ≤ 427 ppm (using the distinctions of the IPCC). Atmospheric CO_2_ rose by 2.5 ppm from 2017 to 2018, if this rate is sustained, our new data indicate that CO_2_ will surpass even the highest mPWP values within the next 5 to 6 years (2025–2026).

## Conclusions

Our new δ^11^B-data for the mPWP provide a tightly constrained and robust estimate of CO_2_ during KM5c of ${391}_{-28}^{+30}$ ppm (at 95% confidence**)**, and documents CO_2_ variability during the mPWP from ${330}_{-21}^{+14}$ to ${391}_{-9}^{+34}$ ppm. This extended view suggests that changes in ocean carbon storage may play an important role in natural variability in CO_2_ on orbital timescales in warmer than present climate states. By better constraining the upper levels of CO_2_ during the mPWP we conclude that, given current rates of CO_2_ increase, we will very likely surpass mPWP values within 4 to 5 years, meaning that, by 2024/2025 levels of atmospheric CO_2_ will be higher than any point in the last 3.3 million years.

## Methods

ODP Site 999 is located in the Caribbean Sea and has been reliably used to reconstruct past atmospheric CO_2_ in a number of previous studies^18,51,52^. Air-sea disequilibrium for CO_2_ in the surface waters in the region of ODP Site 999 are close to 0 (+20 ppm) today and ref. ^13^ suggest this remained the case for at least the last 3.3 million years. An age model for the interval 3.15 to 3.35 Ma studied here was constructed by analyzing the epibenthic foraminifera *Cibicidoides wuellerstorfi* for δ^18^O in each sample, combining these data with similar data from ref. ^53^, and tuning the combined record to the LR04 benthic δ^18^O stack^19^ (Fig. 1). From each sample, ~120 individual tests of the mixed layer dwelling species *Globigerinoides ruber* white *sensu stricto* (300–355 μm) were hand separated, clay removed, oxidatively cleaned and analyzed for boron isotopic (δ^11^B) and trace element composition (e.g. Mg/Ca, Al/Ca) at the University of Southampton using well-established procedures^21,52^. These data were converted to pH using the *G. ruber* δ^11^B-pH calibration of ref. ^51^ and a modern δ^11^B of seawater^54^ (39.6‰). Sea surface temperatures (SST) were derived from each samples Mg/Ca ratio using the calibrations of ref. ^55^ corrected for the changing Mg/Ca for seawater following ref. ^20^ based on ref. ^56^.

It was recently suggested^17^ that the δ^11^B-pH calibration for *G. ruber* may have varied over the last 3.5 million years. We therefore also analyse *T. sacculifer* (300–355 μm) from two samples from ODP 999 to combine with existing *T. sacculifer* data^14,20,52^ to provide a test of the *G. ruber* δ^11^B-pH calibration of ref. ^51^. Following previous studies^13^, uncertainty relating to the following factors, including uncertainty in the δ^11^B-pH calibration, were propagated using a Monte Carlo approach (n = 10,000): temperature (±1.5 °C; 2σ), salinity (± 3 psu, 2σ), δ^11^B_sw_ (± 0.2, 2σ; refs. ^20,57^), analytical uncertainty (± 0.12–0.3‰, 2σ; see Supplementary Table 2).

In order to calculate atmospheric CO_2_ from the reconstructed surface water pH we use dissolved inorganic carbon (DIC) from the reconstructions of ref. ^20^. Uncertainty in this term is also propagated into CO_2_ uncertainty using a Monte Carlo approach (n = 10,000), but with a uniform distribution encompassing the range of the reconstructed DIC for our study interval (1765 to 1840 μmol/kg). CO_2_ was then determined by the maximum probability of all 10,000 realisations.

To obtain CO_2_ during the KM5c interglacial the mean and associated uncertainty of the reconstructed borate ion δ^11^B and SST of the data lying within +/− 7 kyr of the peak of KM5c (n = 5) was determined. In our age model we defined that the peak of KM5c was at 3212 kyr, although expanding the window to ±15 kyr, or uncertainty in the identification of the peak of KM5c to within 10 kyr, did not have a significant effect on our calculated mean (Supplementary Table 1).

This average was then used to calculate pH and CO_2_, but only considering the uncertainty in δ^11^B_sw_ and DIC detailed above. This approach was chosen because it allows the random uncertainties arising from SST and δ^11^B measurement error to be reduced through replication, whilst still realistically accounting for the systematic uncertainties in δ^11^B_sw_ and DIC.

In order to place our new data into a Plio-Pleistocene context, we recalculated the δ^11^B data of ref. ^13^ from 3.0 to 3.3 Ma using the same methodology outlined here and combined it with our new data. The difference in the method is the choice of the second carbonate parameter where constant modern alkalinity is used in ref. ^13^ whereas we use DIC^20^ in this study. These new and published data are then combined into a single record with an average temporal resolution of 1 sample every 4 kyr. This was then interpolated to 1 kyr resolution and a 6-point running mean was used to reduce the influence of analytical uncertainty on our reconstructed CO_2_ record. Uncertainty in the smoothed record was determined using the output of the original Monte Carlo simulation.

CO_2_ forcing was calculated relatively to preindustrial CO_2_ (278 ppm) and is defined as:

$$({\text{R}}_{{\mathrm{CO}}_{2}})=\text{ln}\frac{C{O}_{2}}{C{O}_{2preind}}$$

## Supplementary information

Supplementary figures and table S1

Supplementary table 2
