# Author Correction: Atmospheric CO_2_ during the Mid-Piacenzian Warm Period and the M2 glaciation

**DOI:** 10.1038/s41598-021-93282-w

**Published:** 2021-06-30

**Authors:** Elwyn de la Vega, Thomas B. Chalk, Paul A. Wilson, Ratna Priya Bysani, Gavin L. Foster

**Affiliations:** School of Ocean and Earth Science, University of Southampton, National Oceanography Centre Southampton, Waterfront Campus Southampton, Southampton, SO14 3ZH UK

Correction to: *Scientific Reports* https://doi.org/10.1038/s41598-020-67154-8, published online 09 July 2020

The original version of this Article contained errors.

The data presented in de la Vega, E., Chalk, T.B., Wilson, P.A. *et al.* (2020) is based on a combination of δ^11^B-derived CO_2_ proxy data from Martínez-Botí, M. *et al.* (2015)^1^ and newly generated data, both from contiguous material gathered from ODP Site 999 in the Caribbean Sea.

The original version of this Article contains an error where the CO_2_ data reported from Martínez-Botí, M. *et al.* (2015) was plotted and calculated on its original age model as published. However, the newly generated high resolution δ^11^B/CO_2_ data was plotted and calculated on an updated age model, leading to a small number of points falling out of strict stratigraphic sequence.

The age model of the data from Martínez-Botí, M. *et al.* (2015) has now been updated to our new age model and has been corrected in all Figures, calculations, and Tables affected. This has impacted Figures 1 and 2, the Supplementary Figures 2, 3, 5, 6 and 7, and Supplementary Table 1, 2 and 3. The original Figures 1 and 2, and Supplementary Information file are provided below.

**Figure 1:**
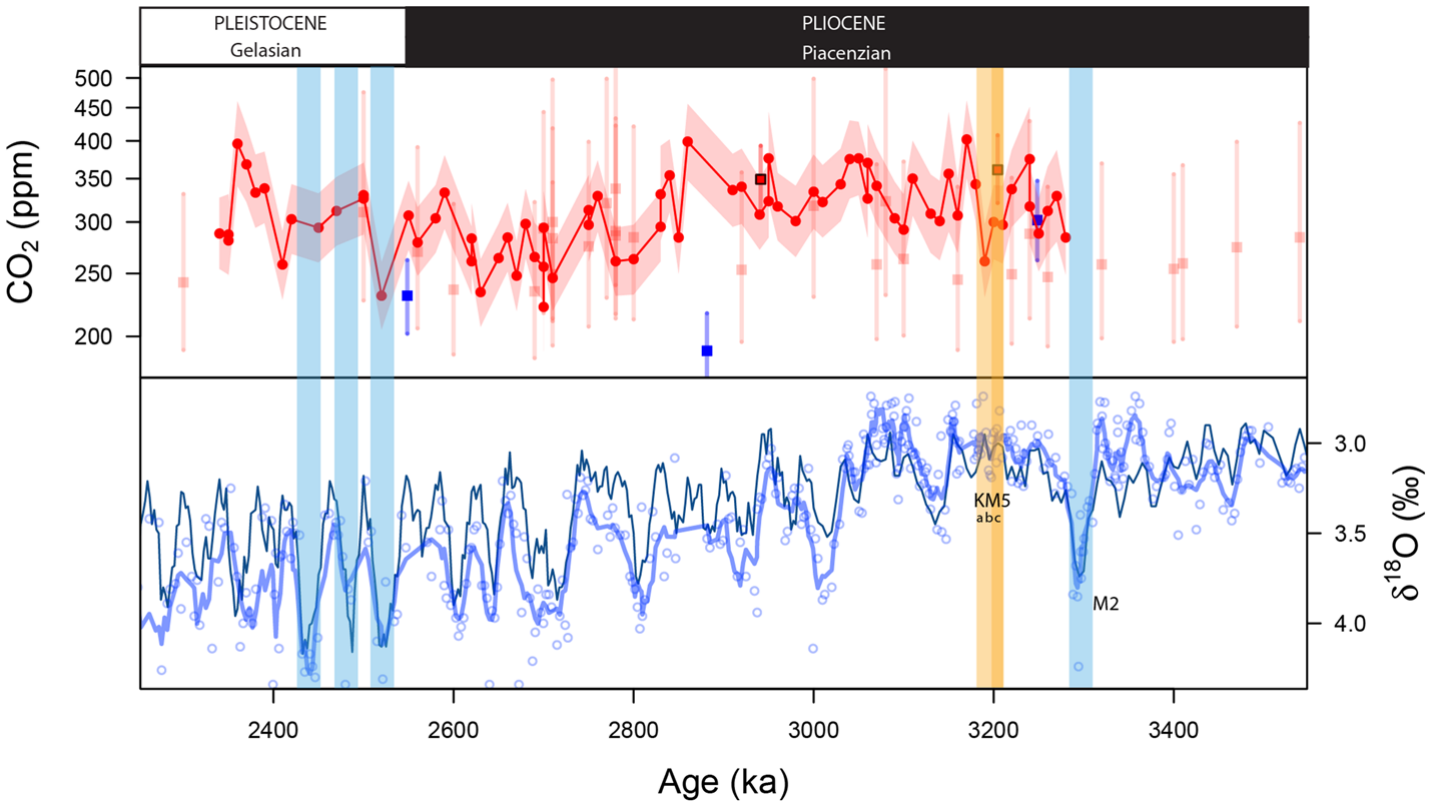
Top panel: Current CO_2_ estimates from boron isotopes across the Plio-Pleistocene boundary. *G. ruber* data in red circles from site 999 (Martinez-Boti *et al.*^13^), *T. sacculifer* in red squares from site 999 (Seki *et al.*^14^), blue squares from site 926 (Sosdian *et al.*^20^) and pale red squares from site 999 (Bartoli *et al.*^12^). Bottom Panel: δ^18^O from benthic foraminifer *Cibicidoides wuellerstorfi* at ODP Site 999 (blue circles) with a 5 point running mean (this study and ref.^53^) compared to the benthic isotope stack of ref.^19^. M2 glacial and early Pleistocene strong glacials are highlighted in blue for context. Interglacial KM5 and KM5c are highlighted in yellow and orange, respectively. Note that there are no estimates for the M2 glacial and very few across the mid-Piacenzian warm period (mPWP), the low resolution of previous studies makes pin-pointing individual interglacials such as the KM5c future analogue difficult. These studies also differ in their estimates of Mid-Piacenzian CO_2_^12,13,14,20^.

**Figure 2:**
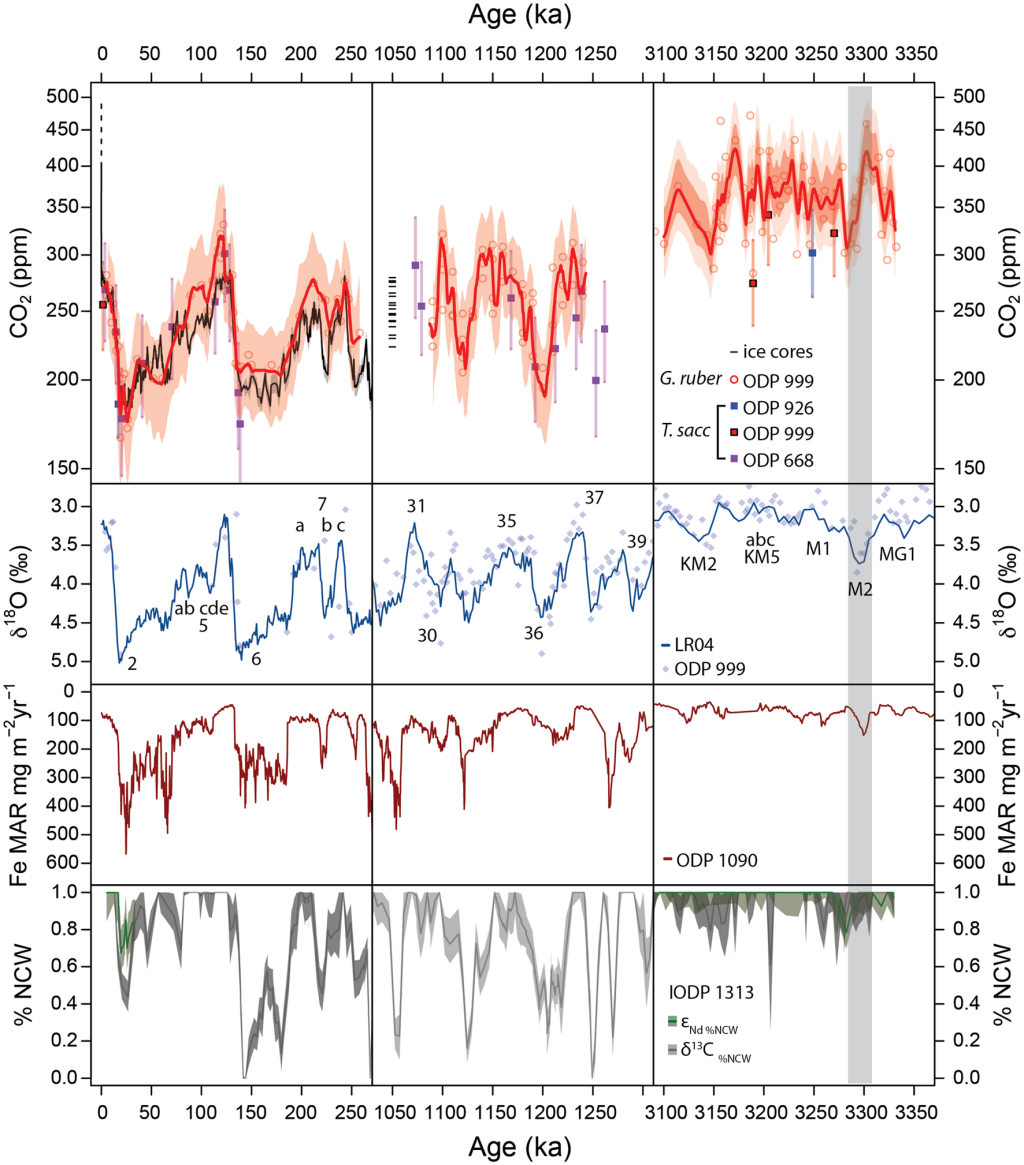
Top panel: Red circles and lines show δ^11^B-derived CO_2_ data from *Globigerinoides ruber* at ODP Site 999 (this study and Martinez-Boti *et al.*^13^, Chalk *et al.*^18^), red squares are *Trilobatus sacculifer* at ODP 999 (this study and Seki *et al.*^14^), purple squares are *T. sacculifer* from ODP 668 (Honisch *et al.*^23^) and blue squares are *T. sacculifer* from ODP 926 (Sosdian *et al.*^20^). Black solid line shows ice core-derived CO_2_ from ref.^58^. Left; Late Pleistocene CO_2_ from boron isotopes^14,18,23,52^ and ice core data. Also shown are CO_2_ projections in line with RCP8.5 at current emission rates to the year 2040 (black broken line). Middle column; MPT CO_2_ estimates^18,23^ including disturbed ice estimates^24,25^ (Note: age adjusted for scale). Right; mPWP estimates of CO_2_ (this study combined with Martinez-Boti *et al.*^13^), new data from *T. sacculifer* is shown in red squares and shows no offset from *G. ruber* estimates. Second panel: Time periods as above, LR04 and ODP 999 δ^18^O from *C. wuellerstorfi*^18,19,53^. Third panel: Iron mass accumulation rate from the Southern Atlantic ODP Site 1090^28^. Fourth panel: % Northern Component Water (NCW) estimated from δ^13^C in benthic foraminifera (grey) and ɛ_Nd_ from fish debris (dark green) in the deep North Atlantic (core U1313^31^). Note the lag of ocean circulation and CO_2_ relative to the M2 glaciation^31^.

This change does not impact the overall conclusion of the article, however, the average CO_2_ values during KM5c and the CO_2_ range during the specific intervals of the study slightly vary from the update.

The original Article and accompanying Supplementary Information files have been corrected.

## Supplementary information

Supplementary Informations.
