# Supplementary material for: Atmospheric CO2 during the Mid-Piacenzian Warm Period and the M2 glaciation
Source: Sci Rep. 2020 Jul 9;10:11002. doi: 10.1038/s41598-020-67154-8 (PMC7347535; doi:10.1038/s41598-020-67154-8)
Supplement: Supplementary file 1 — Supplementary figures and table S1 [file 41598_2020_67154_MOESM1_ESM.docx]

Supplementary Material for

“Atmospheric CO_2_ during the Mid-Piacenzian Warm Period and the M2 glaciation”.

**Elwyn de la Vega^1*^, Thomas B. Chalk^1^, Paul A. Wilson^1^, Ratna Priya Bysani^1^ and Gavin L. Foster^1^**

*^1^School of Ocean and Earth Science, University of Southampton, National Oceanography Centre Southampton, Waterfront Campus Southampton, SO14 3ZH*

**corresponding author:* [*elwyn.de-la-vega@soton.ac.uk*](mailto:elwyn.de-la-vega@soton.ac.uk)

**
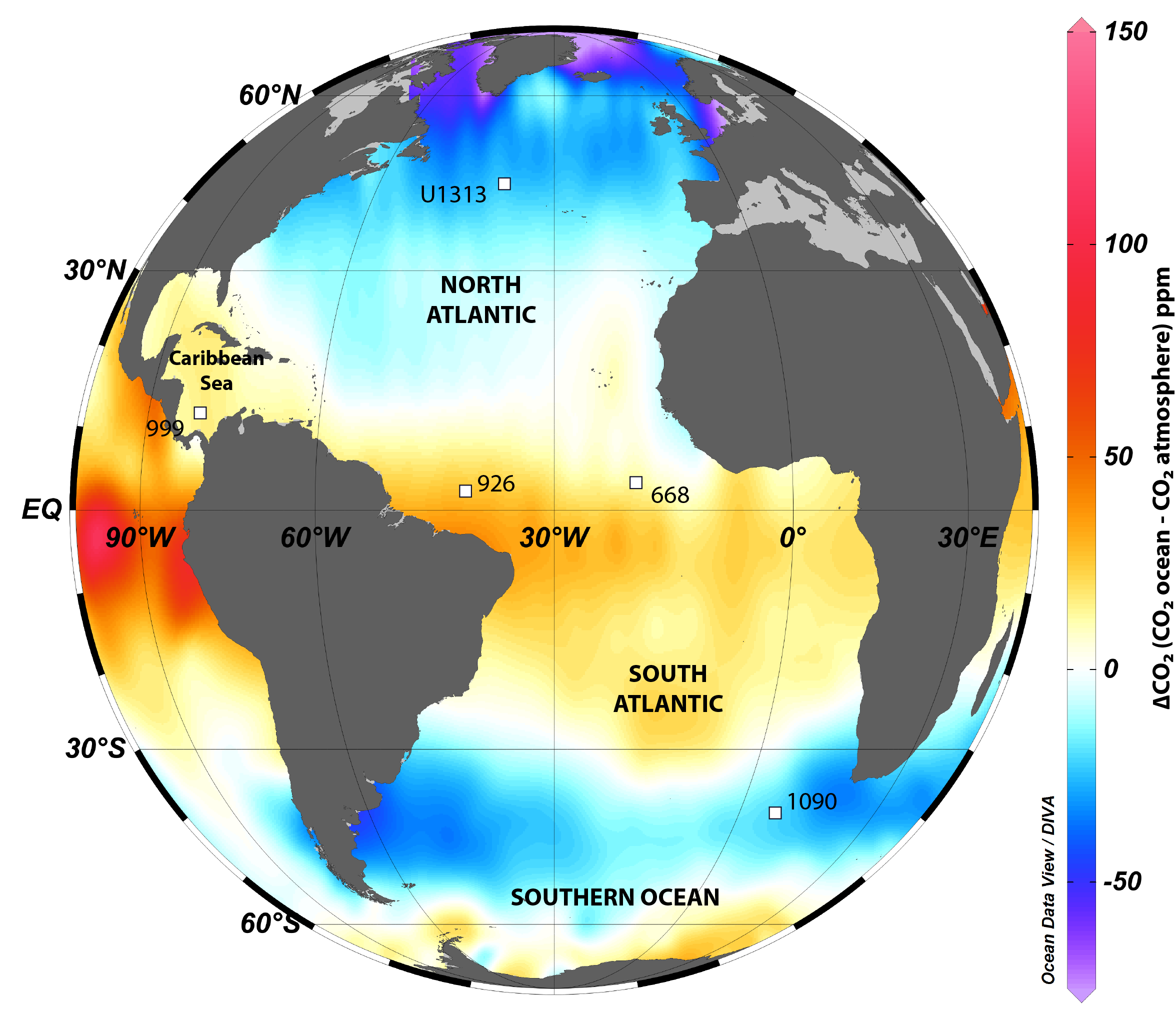
**

**Supplementary Fig 1.** Location of (I)ODP sediment cores mentioned in this study plotted on a map of air-sea CO_2_ disequilibrium (ocean minus atmosphere in parts per million). Warm colours denote areas of CO_2_ source to the atmosphere, and cold colours denote areas of oceanic CO_2_ sink. Site ODP 999 is shown in the Caribbean Sea and has a modern disequilibrium of +21 ppm. Figure made with Ocean Data View.

**Supplementary Fig 2.** Pliocene δ^11^B-derived CO_2_ data with unsmoothed (black) and smoothed (red) lines. Benthic δ^18^O from *Cibicidoides wuellestorfi* (ODP 999, open blue dots and smoothed line) and LR04 benthic δ^18^O stack^19^ (black). Note the ODP 999 δ^18^O data are corrected by 0.64‰ to account for species and machine offsets.

**Supplementary Fig 3.** Comparison between δ^11^B of borate, pH and CO_2_ of *G. ruber* and *T.sacculifer* (without final chamber), showing a good agreement between both species. A 1:1 line is shown in red in each figure.


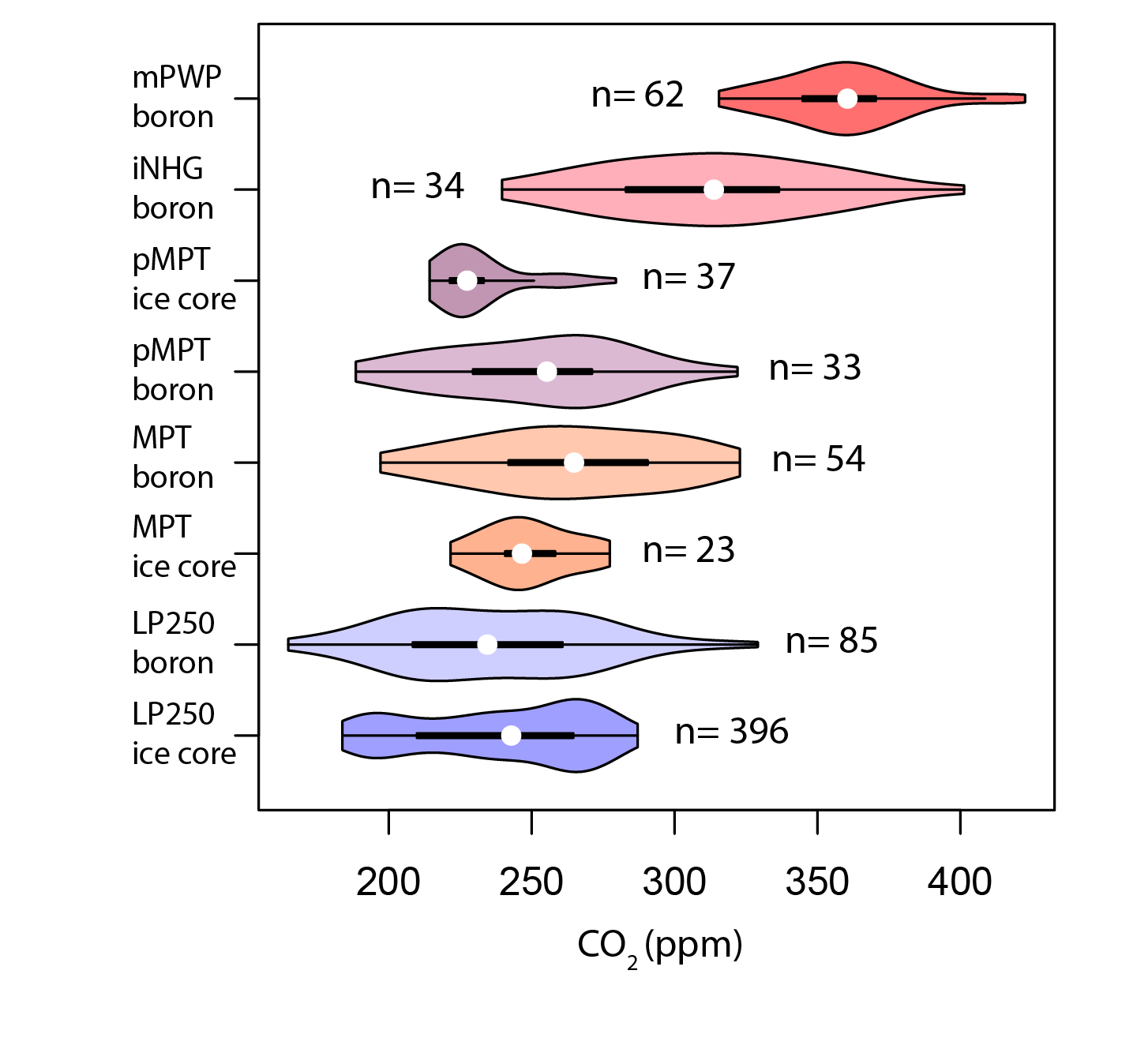


**Supplementary Fig 4.** As Figure 3. Comparison of CO_2_ distributions from ice core record and boron isotopes proxies. From bottom to top: Late Pleistocene (LP, 0-250 ka) ice core (Bereiter et al.^58^) and boron isotopes (Chalk et al.^18^, Honisch et al.^23^, Henehan et al.^51^), Mid-Pleistocene transition (MPT) disturbed ice (Yan et al.^25^) and MPT boron isotopes (Chalk et al.^18^), pre-MPT (pMPT) boron (Dyez et al.^17^) and pMPT disturbed ice (Yan et al.^25^), Plio-Pleistocene intensification of Northern Hemisphere Glaciation (iNHG)^13^, Mid-Piacenzian warm period (mPWP, this study and Martinez-Boti et al.^13^).The number of observations making up the distributions is shown next to the plots.

**
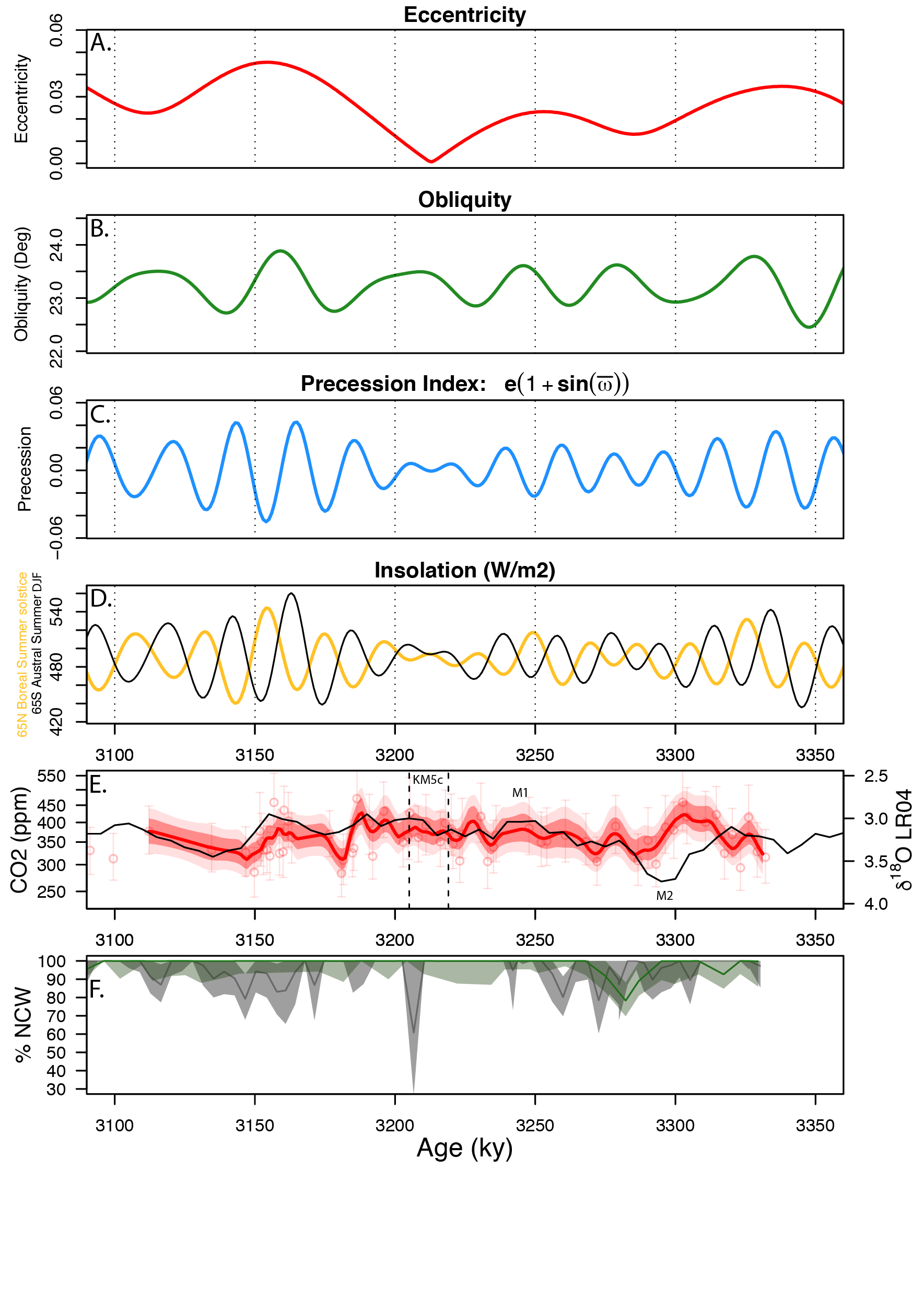
**

**Supplementary Fig 5.** (A-D) Orbital parameters^39^ during the mid-Pliocene warm period, eccentricity, obliquity, precession index and insolation at 65°N (yellow, boreal summer solstice) and 65°S (black, austral summer, average of December, January and February). (E) boron-derived CO_2_ across M2 and the mPWP (red), and LRO4 benthic stack (black). (F) Percentage northern component water determined from Nd isotopes from fish teeth (green) and C isotopes from benthic foraminifera (grey)^31^.


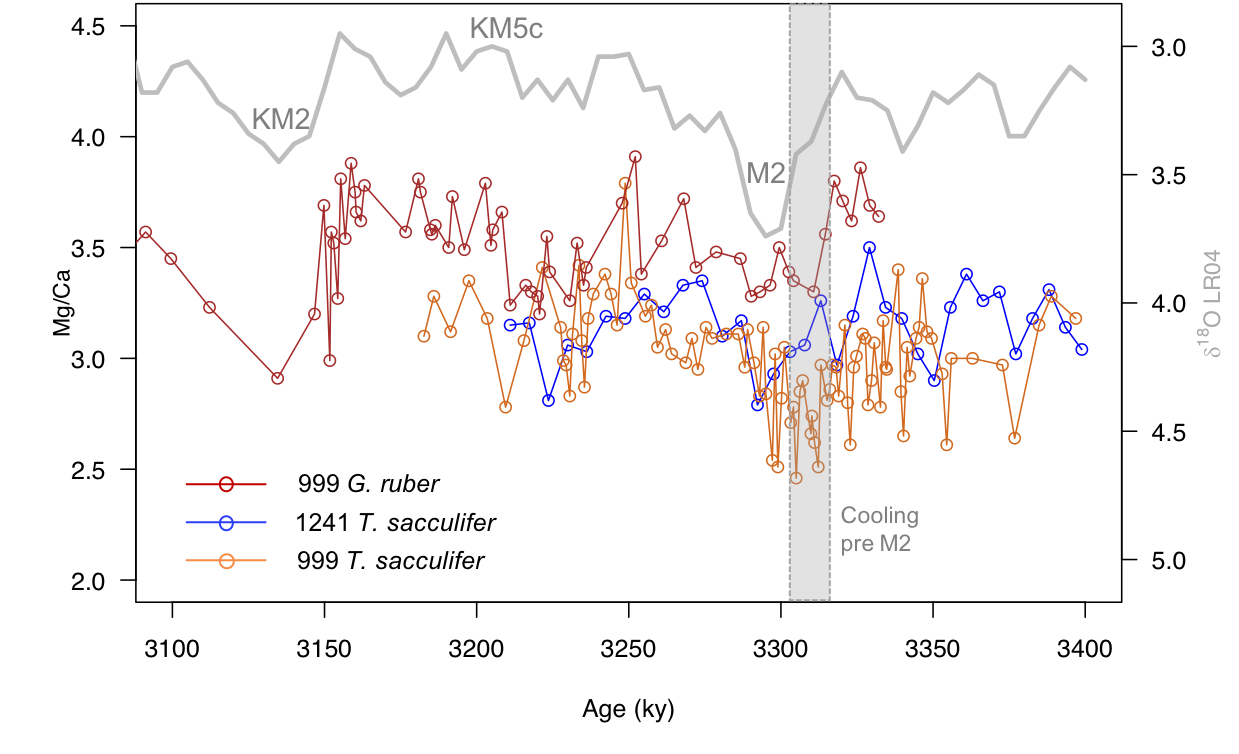


**Supplementary Fig 6.** Mg/Ca (mmol/mol) of *G. ruber* (red, this study) at Caribbean ODP Site 999 and *T. sacculifer* at East Equatorial Pacific ODP Site 1241^48^ (blue) and ODP 999^34^ (orange). LR04 benthic stack^19^ is shown in grey. Mg/Ca of *T. sacculifer* and *G. ruber* at ODP 999 show a cooling at M2 inception before an increase in δ^18^O. The age model of *T. sacculifer* ^34^ has been rescaled to our new age model for consistency.

**
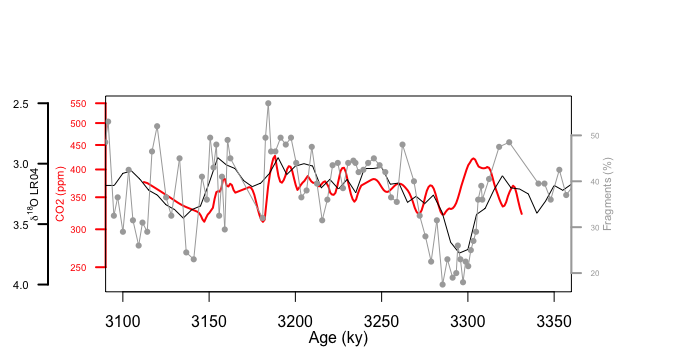
**

**Supplementary Fig 7.** δ^11^B-derived CO_2_ during the mPWP (red line) and percentage fragments^45^ (grey) from the same core (ODP 999). LR04 benthic stack^19^ in black. The age model of ref^45^ has been rescaled to our age model for consistency.

**Supplementary Table 1.** Average CO_2_ for different interval length around KM5c (centered at 3212 ky).

| Length of interval around KM5c | Number of points averaged (n) | Average CO_2_ (ppm) |
| --- | --- | --- |
| ±7 ky | 5 | ${391}_{-28}^{+30}$ |
| ±10 ky | 9 | ${378}_{-25}^{+27}$ |
| ±15 ky | 11 | ${371}_{-25}^{+26}$ |
