# Supplementary material for: Author Correction: Atmospheric CO2 during the Mid-Piacenzian Warm Period and the M2 glaciation
Source: Sci Rep. 2021 Jun 30;11:13920. doi: 10.1038/s41598-021-93282-w (PMC8245415; doi:10.1038/s41598-021-93282-w)
Supplement: Supplementary file 1 — Supplementary Informations. [file 41598_2021_93282_MOESM1_ESM.pdf]

Supplementary Material for

"Atmospheric CO_2 during the Mid-Piacenzian Warm Period and the M2 glaciation".

Elwyn de la Vega[1*], Thomas B. Chalk[1], Paul A. Wilson[1], Ratna Priya Bysani[1] and Gavin L. Foster[1]

[1]School of Ocean and Earth Science, University of Southampton, National Oceanography Centre Southampton, Waterfront Campus Southampton, SO14 3ZH

**corresponding author: elwyn.de-la-vega@soton.ac.uk*

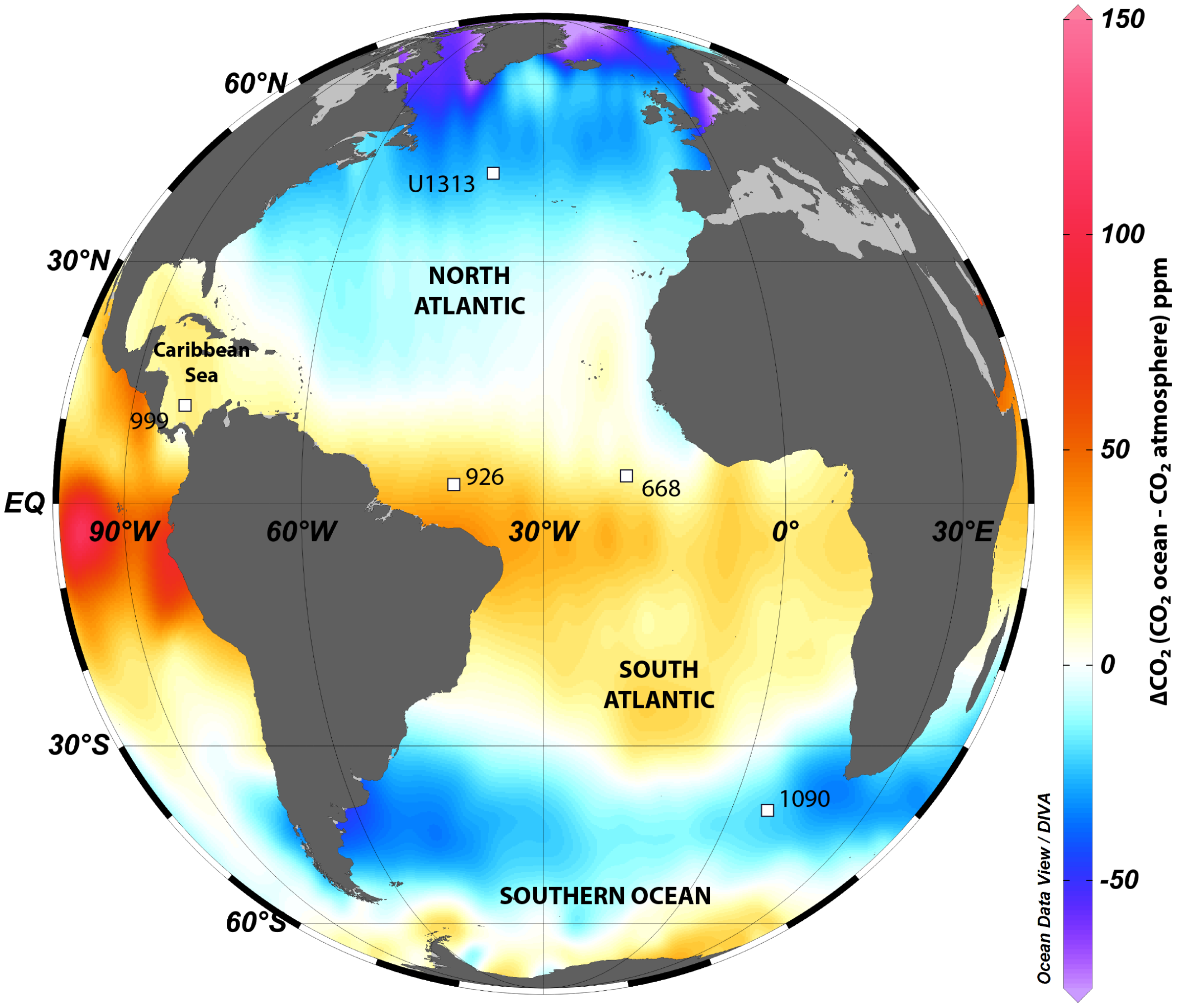

Supplementary Fig 1. Location of (I)ODP sediment cores mentioned in this study plotted on a map of air-sea CO_2 disequilibrium (ocean minus atmosphere in parts per million). Warm colours denote areas of CO_2 source to the atmosphere, and cold colours denote areas of oceanic CO_2 sink. Site ODP 999 is shown in the Caribbean Sea and has a modern disequilibrium of +21 ppm. Figure made with Ocean Data View.

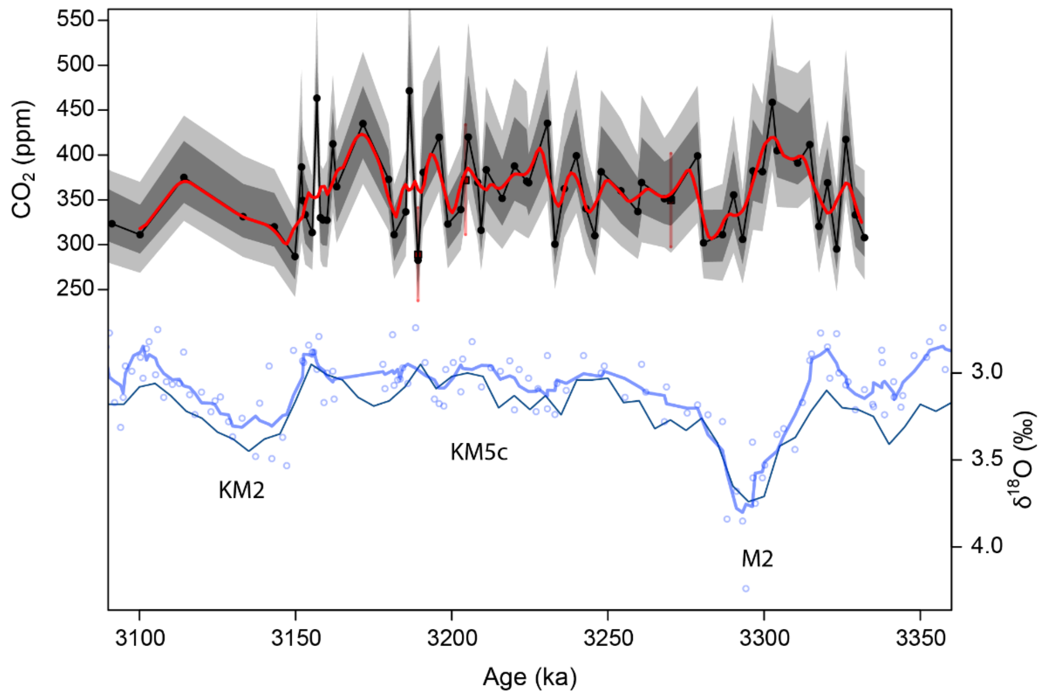

Supplementary Fig 2. Pliocene δ^{11}B-derived CO_2 data with unsmoothed (black) and smoothed (red) lines. Benthic δ^{18}O from *Cibicidoides wuellestorfi* (ODP 999, open blue dots and smoothed line) and LR04 benthic δ^{18}O stack[19] (black). Note the ODP 999 δ^{18}O data are corrected by 0.64‰ to account for species and machine offsets.

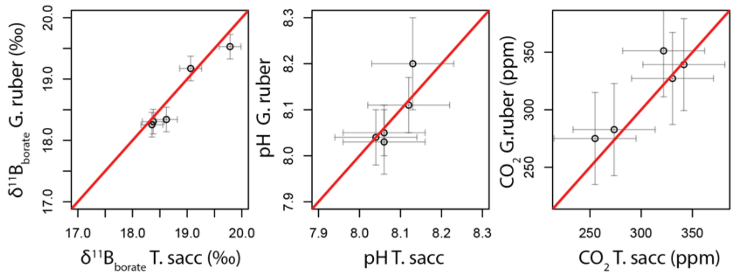

Supplementary Fig 3. Comparison between δ^{11}B of borate, pH and CO_2 of *G. ruber* and *T.sacculifer* (without final chamber), showing a good agreement between both species. A 1:1 line is shown in red in each figure.

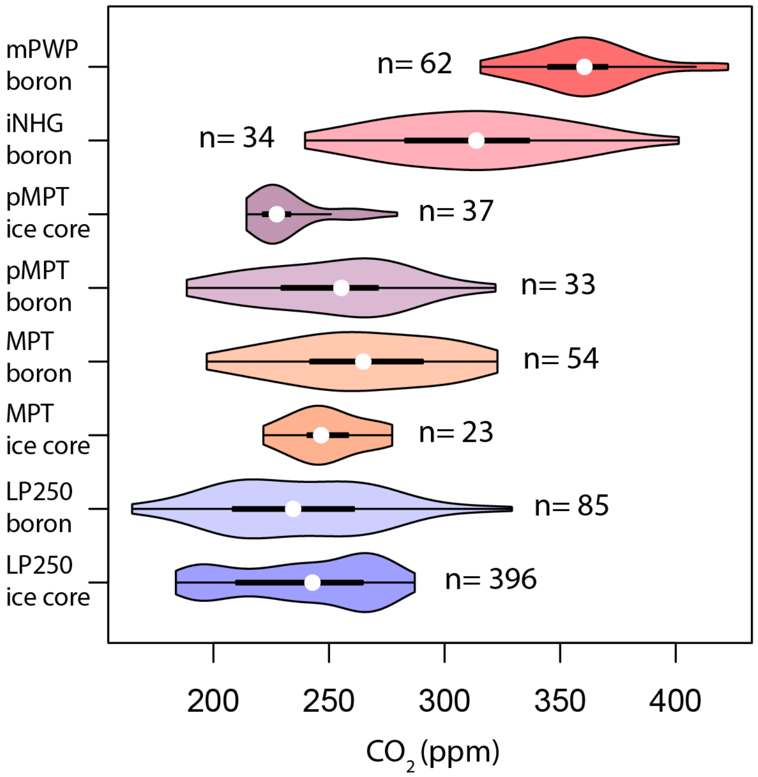

Supplementary Fig 4. As Figure 3. Comparison of CO_2 distributions from ice core record and boron isotopes proxies. From bottom to top: Late Pleistocene (LP, 0-250 ka) ice core (Bereiter et al.[58]) and boron isotopes (Chalk et al.[18], Honisch et al.[23], Henehan et al.[51]), Mid-Pleistocene transition (MPT) disturbed ice (Yan et al.[25]) and MPT boron isotopes (Chalk et al.[18]), pre-MPT (pMPT) boron (Dyez et al.[17]) and pMPT disturbed ice (Yan et al.[25]), Plio-Pleistocene intensification of Northern Hemisphere Glaciation (iNHG)[13], Mid-Piacenzian warm period (mPWP, this study and Martinez-Boti et al.[13]).The number of observations making up the distributions is shown next to the plots.

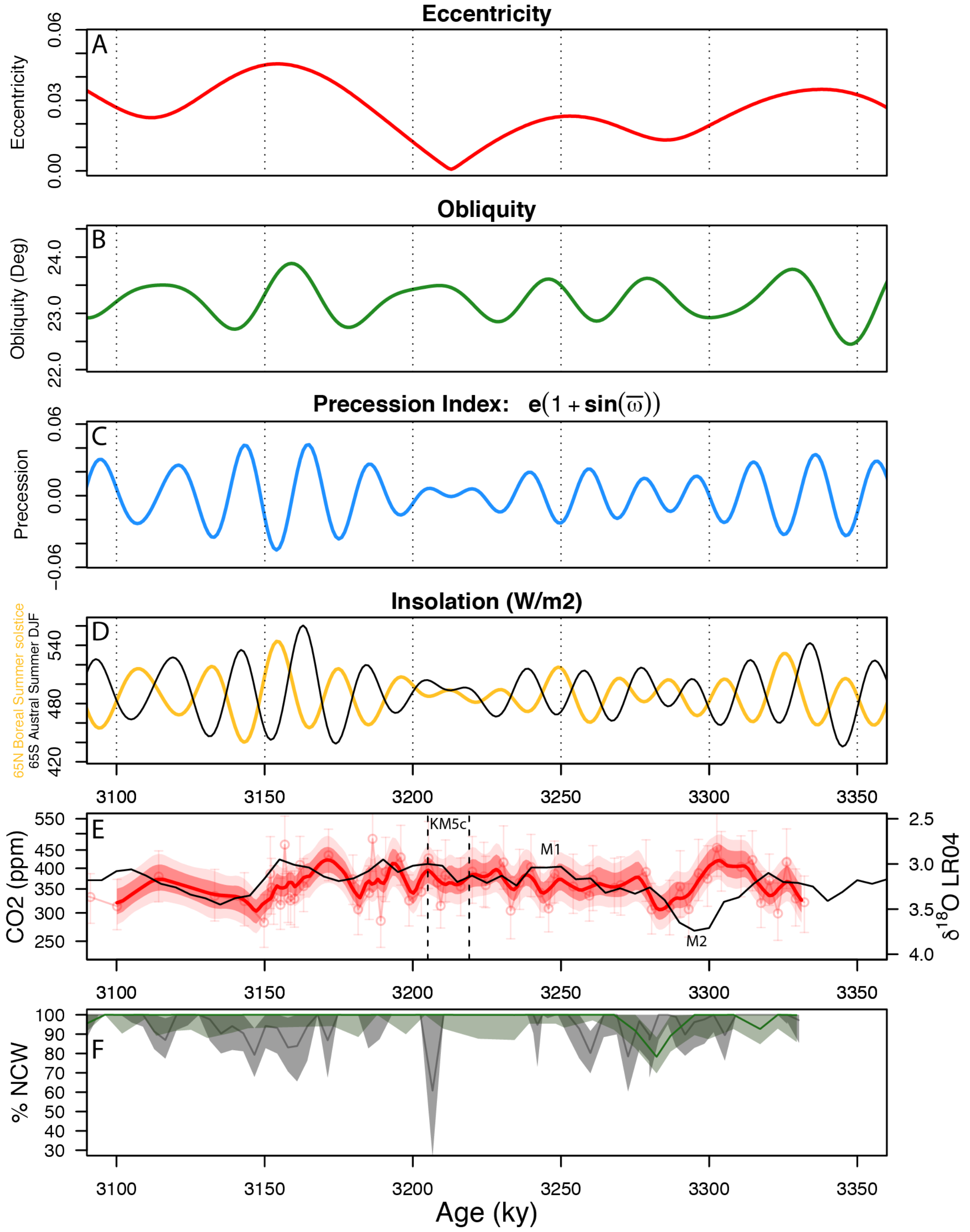

Supplementary Fig 5. (A-D) Orbital parameters[39] during the mid-Pliocene warm period, eccentricity, obliquity, precession index and insolation at 65°N (yellow, boreal summer solstice) and 65°S (black, austral summer, average of December, January and February). (E) boron-derived CO_2 across M2 and the mPWP (red), and LRO4 benthic stack (black). (F) Percentage northern component water determined from Nd isotopes from fish teeth (green) and C isotopes from benthic foraminifera (grey)[31].

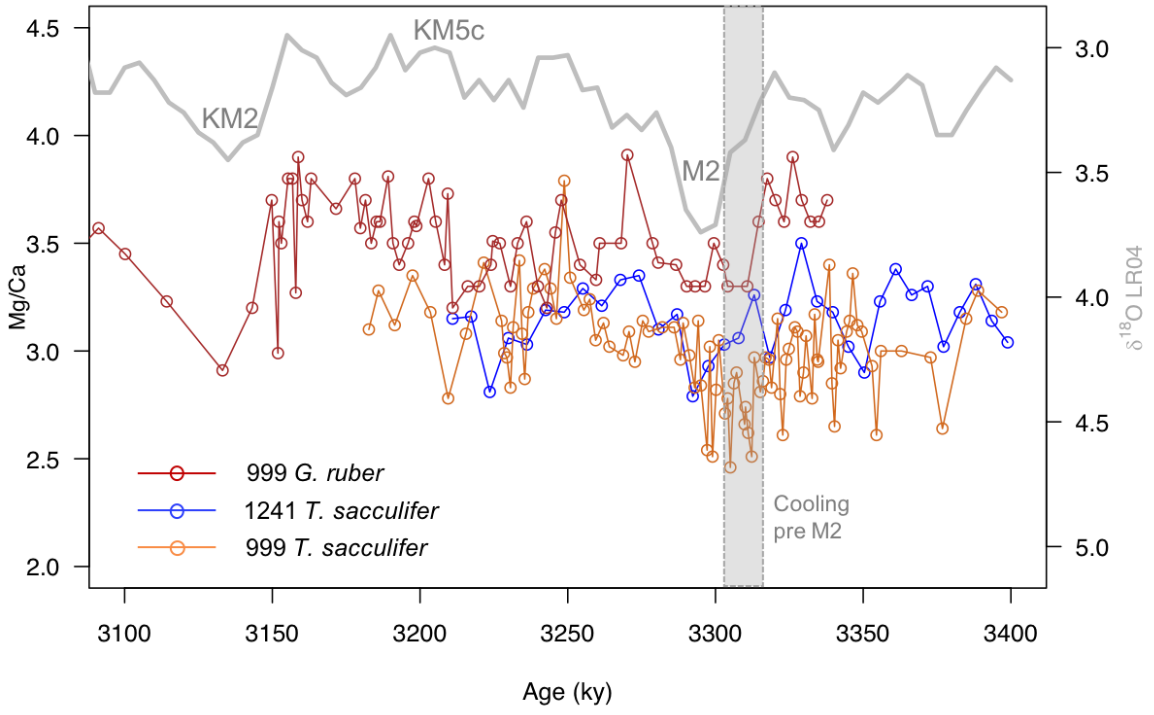

Supplementary Fig 6. Mg/Ca (mmol/mol) of *G. ruber* (red, this study) at Caribbean ODP Site 999 and *T. sacculifer* at East Equatorial Pacific ODP Site 1241[48] (blue) and ODP 999[34] (orange). LR04 benthic stack[19] is shown in grey. Mg/Ca of *T. sacculifer* and *G. ruber* at ODP 999 show a cooling at M2 inception before an increase in δ^{18}O. The age model of *T. sacculifer*[34] has been rescaled to our new age model for consistency.

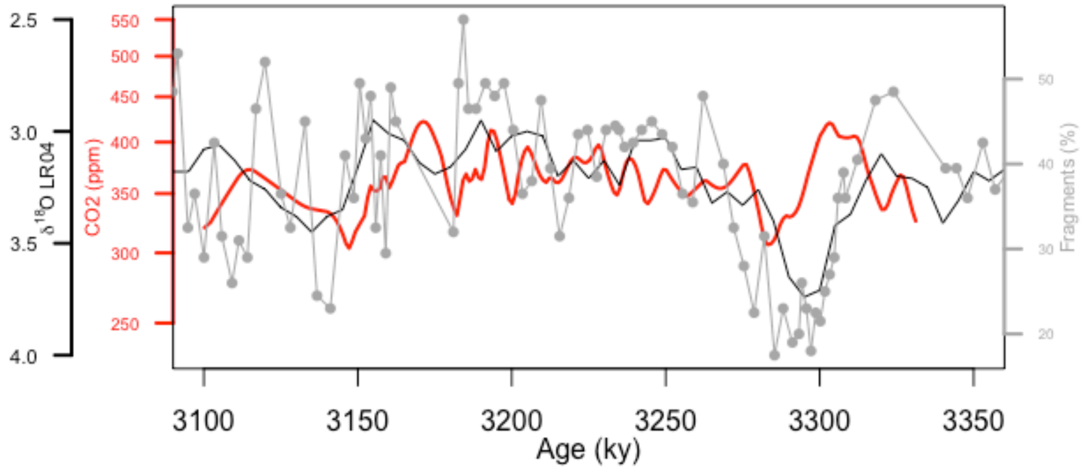

Supplementary Fig 7. δ^{11}B-derived CO_2 during the mPWP (red line) and percentage fragments[45] (grey) from the same core (ODP 999). LR04 benthic stack[19] in black. The age model of ref[45] has been rescaled to our age model for consistency.

Supplementary Table 1. Average CO_2 for different interval length around KM5c (centered at 3212 ky).

Length of interval around KM5c	Number of points averaged (n)	Average CO_2 (ppm)
±7 ky	5	372^{+31}_{-29}
±10 ky	7	371^{+28}_{-26}
±15 ky	10	367^{+26}_{-24}

Supplementary Table 2. δ^{11}B data and 2sd uncertainty, and boron-derived CO_2 calculated during the mPWP including published data (Martinez-Boti et al.[13]) and new data (this study), see supplementary table 3 for reference to ID. Uncertainty values are indicated at 68% confidence (lw68 and up68, lower and upper bound) and at 95% confidence (lw95 and up95, lower and upper bound). pCO2.av and pCO2.max denote average and maximum probability CO_2.

Age (ky)	pCO2.av	pCO2.max	lw95	lw68	up68	up95	δ^{11}B	2sd
2337.82	311.72	317.73	271.28	283.08	335.31	361.38	20.23	0.10
2346.71	311.36	306.25	261.36	282.34	342.74	364.33	20.21	0.18
2352.72	302.54	293.50	254.48	277.24	331.33	354.00	20.40	0.11
2372.20	391.11	383.86	340.57	357.37	429.75	444.85	19.65	0.12
2381.29	358.07	365.63	290.27	320.29	391.38	414.99	19.89	0.12
2389.32	367.79	377.28	305.54	330.99	401.30	428.87	19.83	0.10
2410.92	275.33	282.34	232.50	248.93	301.24	320.55	20.57	0.09
2424.89	326.63	331.23	278.12	294.32	355.41	379.57	20.10	0.10

2451.23	315.30	305.86	256.78	285.08	349.74	367.59	20.20	0.09
2474.49	334.92	331.81	283.26	307.99	361.49	392.99	20.04	0.09
2500.40	351.33	352.69	305.40	316.69	380.33	411.44	19.91	0.11
2501.40	358.50	350.33	308.96	329.36	393.97	412.98	19.88	0.09
2521.93	249.23	247.50	209.30	229.40	268.28	283.35	20.71	0.09
2545.15	328.13	334.39	276.21	299.48	361.10	379.27	20.00	0.09
2561.90	300.53	295.43	264.05	277.61	323.99	339.38	20.32	0.10
2579.43	328.47	339.42	280.56	296.65	355.78	383.10	20.04	0.10
2594.84	355.93	369.45	293.28	319.81	389.15	411.38	19.93	0.13
2615.07	277.38	290.43	235.94	251.50	301.09	317.23	20.42	0.09
2624.50	303.60	304.38	252.01	280.63	323.81	350.54	20.23	0.10
2630.91	250.21	254.65	210.34	226.82	269.95	291.09	20.80	0.10
2648.26	283.57	276.11	243.70	261.23	307.84	318.30	20.43	0.09
2664.70	300.79	313.64	252.86	267.88	325.02	347.61	20.36	0.11
2673.02	262.27	256.66	227.33	241.60	284.10	298.06	20.58	0.11
2680.36	316.53	317.37	271.95	289.33	339.45	355.36	20.04	0.09
2686.56	283.80	289.12	241.64	260.22	305.20	333.04	20.31	0.10
2695.16	276.55	264.43	235.56	252.03	300.26	320.93	20.35	0.09
2700.04	237.75	230.58	196.82	213.85	262.66	276.26	20.75	0.22
2703.20	317.68	313.31	274.59	290.66	346.62	369.06	20.05	0.13
2707.21	266.27	264.15	227.01	244.93	287.65	305.74	20.51	0.09
2745.52	336.56	337.63	279.86	309.34	364.42	388.50	19.99	0.09
2751.48	318.78	325.57	272.08	288.70	349.04	375.02	20.11	0.15
2764.19	350.30	340.01	296.58	322.18	384.85	402.95	19.86	0.11
2777.48	277.04	271.16	239.19	258.60	303.48	321.17	20.37	0.09
2798.54	282.71	288.47	245.43	258.16	304.87	322.72	20.40	0.11
2830.18	315.58	305.91	258.58	287.39	344.88	367.83	20.20	0.11
2831.68	358.53	361.31	298.45	319.65	389.61	422.89	19.87	0.24
2838.90	377.31	366.72	329.70	346.16	409.99	437.42	19.67	0.10
2849.18	307.26	299.00	254.60	273.04	338.98	366.72	20.16	0.11
2858.32	426.55	404.93	364.38	389.20	466.86	503.33	19.31	0.10
2912.38	358.25	363.72	302.50	323.75	388.45	417.67	19.70	0.10
2923.56	365.09	343.84	314.52	332.72	396.59	424.71	19.66	0.10
2941.58	329.99	321.83	280.48	305.26	361.20	374.81	19.97	0.09
2950.49	346.28	348.17	298.57	317.13	379.31	406.06	19.76	0.11
2951.16	404.33	402.42	345.83	364.34	441.62	496.93	19.46	0.20
2959.60	339.39	358.12	286.02	309.67	369.88	390.06	20.01	0.09
2978.07	324.45	318.28	268.70	287.39	355.94	378.54	20.02	0.09
3002.81	356.63	355.74	297.93	319.70	391.78	405.83	19.74	0.10
3011.00	348.90	366.34	298.83	317.32	378.85	396.92	19.79	0.11
3027.58	368.68	358.30	311.46	337.36	404.17	428.80	19.65	0.10
3042.66	401.33	396.73	345.03	362.79	439.60	465.97	19.46	0.13

3050.02	400.68	404.12	346.78	367.27	428.98	456.10	19.58	0.09
3059.03	351.27	337.38	298.78	323.69	382.79	407.45	19.84	0.15
3062.96	397.28	391.18	326.73	361.32	434.85	478.91	19.52	0.11
3066.54	367.49	351.12	311.24	334.80	402.00	424.69	19.67	0.11
3091.18	327.51	330.32	279.92	301.96	353.72	371.49	20.03	0.11
3100.18	310.58	307.79	270.39	281.25	338.97	356.13	20.10	0.11
3114.24	380.73	362.50	330.43	349.68	416.71	454.26	19.61	0.10
3133.15	335.57	335.02	287.10	309.34	360.16	392.89	19.80	0.13
3143.17	320.81	315.16	270.39	292.02	351.90	376.48	19.95	0.15
3149.80	282.21	287.13	230.69	250.04	307.43	323.89	20.42	0.16
3151.90	385.93	377.91	297.55	330.05	438.96	473.71	19.51	0.33
3152.24	352.24	337.40	297.43	313.47	389.80	421.96	19.85	0.19
3153.05	337.37	324.37	268.65	300.77	377.87	412.39	19.97	0.23
3155.33	313.45	302.50	259.94	285.58	341.77	361.23	20.17	0.12
3156.80	462.08	469.41	400.09	418.66	505.08	533.42	19.24	0.18
3157.94	332.82	317.93	277.34	299.13	367.65	395.39	19.93	0.11
3158.75	323.62	328.25	265.95	298.51	350.59	371.18	20.11	0.13
3160.05	329.31	316.35	284.16	302.19	362.30	396.05	20.08	0.20
3161.92	406.15	386.33	335.28	364.23	452.27	485.97	19.55	0.21
3163.14	358.91	371.12	289.07	324.14	390.74	419.14	19.87	0.16
3171.55	432.91	434.40	364.93	392.50	477.26	503.47	19.41	0.11
3179.79	372.75	376.19	314.27	340.76	404.93	441.86	19.75	0.09
3181.53	302.25	305.64	255.41	272.95	330.40	351.26	20.22	0.17
3185.27	339.44	336.81	275.05	303.77	372.71	402.40	19.94	0.16
3186.41	478.96	461.48	384.61	430.30	538.69	572.26	19.15	0.27
3189.19	280.01	287.97	236.94	255.96	305.37	319.67	20.46	0.10
3190.83	394.89	388.77	324.72	355.23	436.98	477.06	19.59	0.27
3195.95	424.52	409.87	361.44	380.28	468.34	504.70	19.37	0.21
3198.73	319.22	312.55	269.22	292.06	346.60	369.19	20.06	0.12
3202.88	343.86	353.90	293.01	319.47	373.60	391.64	19.95	0.14
3205.30	435.92	415.77	355.68	386.19	489.01	558.23	19.39	0.29
3208.31	363.67	366.32	306.30	325.08	398.28	445.43	19.78	0.23
3209.38	321.34	321.48	270.45	289.23	357.81	382.03	20.12	0.15
3211.02	381.96	375.86	321.46	345.49	418.34	456.52	19.54	0.26
3216.14	348.87	357.32	284.78	317.34	379.69	408.06	19.79	0.22
3220.06	389.40	364.42	317.40	348.76	433.77	462.34	19.54	0.29
3223.98	379.35	374.62	310.47	334.80	421.96	467.87	19.63	0.27
3224.60	364.73	369.94	306.31	336.04	388.13	434.94	19.77	0.13
3230.61	422.85	402.19	349.37	371.70	471.96	519.37	19.36	0.24
3233.02	304.47	301.15	250.31	274.01	332.84	365.68	20.17	0.26
3236.04	364.82	354.18	305.91	333.24	401.77	435.11	19.71	0.24
3239.89	412.63	407.21	339.58	369.99	460.93	489.99	19.47	0.25

3242.93	340.61	338.26	293.57	312.63	372.22	396.06	19.83	0.11
3245.81	310.63	313.07	265.49	286.57	339.92	357.78	20.16	0.10
3247.82	394.18	394.09	326.67	357.54	437.92	485.62	19.66	0.25
3254.13	354.41	357.55	290.06	319.11	394.29	417.84	19.76	0.24
3259.58	331.13	339.99	279.67	298.12	359.57	399.36	19.90	0.19
3260.77	372.38	380.59	299.72	328.14	411.24	445.11	19.71	0.28
3268.07	354.63	350.39	305.32	319.98	389.00	419.16	19.87	0.18
3270.17	349.70	350.53	282.94	317.16	390.43	419.39	19.92	0.23
3278.70	393.93	370.22	327.88	353.25	438.50	489.56	19.59	0.25
3280.60	302.47	292.60	261.99	276.65	330.77	351.41	20.15	0.12
3286.67	307.31	299.34	267.79	282.99	331.72	354.87	20.14	0.17
3290.24	360.37	357.57	289.16	316.88	403.23	431.25	19.74	0.29
3293.11	302.48	302.09	258.98	272.52	332.74	348.89	20.14	0.16
3296.43	394.34	381.58	318.10	357.18	436.57	471.79	19.55	0.24
3299.45	384.40	384.95	313.97	353.69	413.75	459.36	19.68	0.23
3302.61	460.72	438.90	379.23	403.10	526.93	557.87	19.21	0.23
3304.12	413.95	412.38	335.80	364.19	462.47	485.72	19.42	0.21
3310.76	408.21	409.62	339.04	375.07	445.35	464.23	19.48	0.23
3314.60	415.32	389.89	355.50	375.52	459.37	498.41	19.46	0.22
3317.52	323.27	323.77	261.79	288.60	351.31	380.73	20.10	0.21
3320.30	369.92	364.73	317.44	332.78	408.99	438.15	19.77	0.14
3323.22	296.17	294.71	252.45	267.27	329.81	354.01	20.25	0.21
3326.14	421.86	409.96	349.06	376.22	471.81	494.03	19.50	0.22
3329.20	327.09	326.21	273.83	293.87	355.90	381.13	20.04	0.22
3332.13	314.17	305.61	266.67	283.49	348.54	374.91	20.13	0.21

Supplementary Table 3. Mg/Ca data of *Globigerinoides ruber (sensu stricto)* white used in the calculation of CO_2 and SST including published data (MB15, Martinez-Boti et al.[13]) and new data (EV, this study). $\delta^{18}O$ of benthic foraminifera *Cibicidoides wuellerstorfi* used for age model constraint from orbital tuning to LR04 $\delta^{18}O$ benthic stack[19].

Age (ky)	$\delta^{18}O$ *C. wuell*	Mg/Ca *G. rub*	SST (°C)	ID
2337.82	NA	3.85	28.41	MB15
2346.71	NA	3.71	28.00	MB15
2352.72	NA	4.31	29.67	MB15
2372.2	NA	3.87	28.48	MB15
2381.29	NA	3.91	28.60	MB15
2389.32	NA	3.8	28.29	MB15
2410.92	NA	4.06	29.03	MB15
2424.89	NA	3.81	28.33	MB15
2451.23	NA	3.85	28.46	MB15

2474.49	NA	3.81	28.36	MB15
2500.4	NA	3.7	28.05	MB15
2501.4	NA	3.69	28.02	MB15
2521.93	NA	3.49	27.41	MB15
2545.15	NA	3.48	27.39	MB15
2561.9	NA	3.8	28.37	MB15
2579.43	NA	3.52	27.52	MB15
2594.84	NA	3.97	28.87	MB15
2615.07	NA	3.53	27.57	MB15
2624.5	NA	3.6	27.79	MB15
2630.91	NA	3.89	28.65	MB15
2648.26	NA	3.67	28.01	MB15
2664.7	NA	4.12	29.31	MB15
2673.02	NA	3.61	27.84	MB15
2680.36	NA	3.34	26.98	MB15
2686.56	NA	3.28	26.78	MB15
2695.16	NA	3.13	26.26	MB15
2700.04	NA	3.21	26.55	MB15
2703.2	NA	3.26	26.72	MB15
2707.21	NA	3.33	26.96	MB15
2745.52	NA	3.6	27.84	MB15
2751.48	NA	3.58	27.78	MB15
2764.19	NA	3.55	27.69	MB15
2777.48	NA	3.34	27.01	MB15
2798.54	NA	3.52	27.60	MB15
2830.18	NA	3.87	28.67	MB15
2831.68	NA	3.68	28.11	MB15
2838.9	NA	3.47	27.46	MB15
2849.18	NA	3.36	27.10	MB15
2858.32	NA	3.13	26.31	MB15
2912.38	NA	3.16	26.41	MB15
2923.56	NA	3.11	26.23	MB15
2941.58	NA	3.43	27.32	MB15
2950.49	NA	3.06	26.05	MB15
2951.16	NA	3.22	26.62	MB15
2959.6	NA	3.86	28.63	MB15
2978.07	NA	3.4	27.22	MB15
3002.81	NA	3.28	26.81	MB15
3011	NA	3.18	26.47	MB15
3027.58	NA	3.18	26.47	MB15
3042.66	NA	3.22	26.60	MB15
3050.02	NA	3.75	28.29	MB15

3059.03	NA	3.46	27.40	MB15
3062.96	NA	3.35	27.04	MB15
3066.54	NA	3.21	26.56	MB15
3091.18	NA	3.57	27.74	MB15
3100.18	NA	3.45	27.36	MB15
3114.24	NA	3.23	26.62	MB15
3133.15	NA	2.91	25.46	MB15
3143.17	NA	3.2	26.52	MB15
3149.8	2.74	3.689	28.10	EV
3151.9	NA	2.99	25.76	MB15
3152.24	2.29	3.5656	27.72	EV
3153.05	2.02	3.5186	27.57	EV
3155.33	2.24	3.8052	28.44	EV
3156.8	2.33	3.5395	27.64	EV
3157.94	NA	3.27	26.76	MB15
3158.75	2.42	3.8809	28.66	EV
3160.05	2.36	3.7498	28.28	EV
3161.92	2.35	3.6188	27.88	EV
3163.14	2.32	3.7787	28.36	EV
3171.55	NA	3.66	28.01	MB15
3179.79	NA	3.57	27.73	MB15
3181.53	2.43	3.7492	28.27	EV
3185.27	2.10	3.5576	27.69	EV
3186.41	2.29	3.6011	27.82	EV
3189.19	NA	3.81	28.45	MB15
3190.83	2.46	3.5045	27.52	EV
3195.95	NA	3.4883	27.47	EV
3198.73	NA	3.58	27.76	MB15
3202.88	2.41	3.794	28.40	EV
3205.3	2.48	3.5848	27.77	EV
3208.31	2.29	3.6579	27.99	EV
3209.38	NA	3.73	28.21	EV
3211.02	2.22	3.2401	26.65	MB15
3216.14	2.31	3.3255	26.93	EV
3220.06	2.57	3.2759	26.77	EV
3223.98	2.49	3.393	27.16	EV
3224.6	NA	3.51	27.53	EV
3230.61	2.57	3.2562	26.70	EV
3233.02	2.50	3.523	27.57	EV
3236.04	2.45	3.4127	27.22	EV
3239.89	NA	3.3	26.85	MB15
3242.93	NA	3.2	26.50	MB15

3245.81	NA	3.55	27.66	MB15
3247.82	2.36	3.6958	28.10	EV
3254.13	2.16	3.382	27.12	EV
3259.58	NA	3.33	26.94	MB15
3260.77	2.31	3.5284	27.59	EV
3268.07	2.64	3.7168	28.16	EV
3270.17	NA	3.91	28.73	MB15
3278.7	2.54	3.4784	27.43	EV
3280.6	NA	3.41	27.20	MB15
3286.67	**2.64**	3.4453	27.32	EV
3290.24	2.55	3.2758	26.76	EV
3293.11	3.21	3.3004	26.84	EV
3296.43	**2.96**	3.325	26.92	EV
3299.45	2.96	3.5039	27.50	EV
3302.61	2.84	3.3922	27.14	EV
3304.12	**2.71**	3.3472	26.99	EV
3310.76	2.50	3.3023	26.84	EV
3314.6	**2.29**	3.5643	27.69	EV
3317.52	NA	3.7993	28.40	EV
3320.3	2.21	3.7102	28.14	EV
3323.22	**2.13**	3.6212	27.87	EV
3326.14	**2.42**	3.8584	28.57	EV
3329.2	**2.57**	3.6932	28.09	EV
3332.13	**2.46**	3.6419	27.93	EV
